# Successive Invasion-Mediated Interspecific Hybridizations and Population Structure in the Endangered Cichlid *Oreochromis mossambicus*

**DOI:** 10.1371/journal.pone.0063880

**Published:** 2013-05-09

**Authors:** Cyril Firmat, Paul Alibert, Michèle Losseau, Jean-François Baroiller, Ulrich K. Schliewen

**Affiliations:** 1 UMR CNRS 6282 Biogéosciences – Université de Bourgogne, Dijon, France; 2 Centre for Biodiversity Dynamics (CBD), Department of Biology, Norwegian University of Science and Technology (NTNU), Trondheim, Norway; 3 Polana Caniço A, Maputo, Mozambique; 4 UMR110 Cirad-Ifremer INTREPID, Montpellier, France; 5 Department of Ichthyology, Bavarian State Collection of Zoology (ZSM), München, Germany; Instituto de Higiene e Medicina Tropical, Portugal

## Abstract

Hybridization between invasive and native species accounts among the major and pernicious threats to biodiversity. The Mozambique tilapia *Oreochromis mossambicus*, a widely used freshwater aquaculture species, is especially imperiled by this phenomenon since it is recognized by the IUCN as an endangered taxon due to genetic admixture with *O. niloticus* an invasive congeneric species. The Lower Limpopo and the intermittent Changane River (Mozambique) drain large wetlands of potentially great importance for conservation of *O. mossambicus*, but their populations have remained unstudied until today. Therefore we aimed (1) to estimate the autochthonous diversity and population structure among genetically pure *O. mossambicus* populations to provide a baseline for the conservation genetics of this endangered species, (2) to quantify and describe genetic variation of the invasive populations and investigate the most likely factors influencing their spread, (3) to identify *O. mossambicus* populations unaffected by hybridization. Bayesian assignment tests based on 423 AFLP loci and the distribution of 36 species-specific mitochondrial haplotypes both indicate a low frequency of invasive and hybrid genotypes throughout the system, but nevertheless reveal evidence for limited expansion of two alien species (*O. niloticus* and *O. andersonii*) and their hybrids in the Lower Limpopo. *O. mossambicus* populations with no traces of hybridization are identified. They exhibit a significant genetic structure. This contrasts with previously published estimates and provides rather promising auspices for the conservation of *O. mossambicus*. Especially, parts of the Upper Changane drainage and surrounding wetlands are identified as refugial zones for *O. mossambicus* populations. They should therefore receive high conservation priority and could represent valuable candidates for the development of aquaculture strains based on local genetic resources.

## Introduction

The increasing human influence on earth ecosystems may cause major alterations of patterns of genetic exchange between populations and species [1]. Translocations of exotic species that hybridize with native ones rank among the most important factors eventually leading to species amalgamation and collapse [2], [3]. This threat therefore provides challenging issues for conservation biologists [4]. Hybridization is recognized as a major driving force in evolutionary biology [5], [6] and the evolutionary potential of hybrid lineages has to be fully considered in a conservation context [7], [8]. Introducing new genetic variation into a system, invasion-mediated hybridization has the potential to promote the success and the expansion of hybrid lineages (e.g. [9], [10]). Occurrence and patterns of hybridization are believed to depend on several factors such as the intensity of selection against the non-native parent, the inbreeding costs of locally adapted native populations [11], behavioral traits including mate choice [12] or the persistence of heterotic effects over hybrid generations [13]. Although theoretically essential for conservation genetics, all above mentioned parameters are difficult to estimate in the field, and conservation practice therefore has often to employ genetic or phenotypic estimates of hybridization patterns as observed in wild populations. Allendorf *et al*. [4] distinguished three categories of invasion-mediated hybridization according to the frequency and the expansion of hybrids within the native species' range: (1) hybridization without genetic introgression, i.e. the hybrids beyond the F_1_ generation are absent; (2) hybridization with widespread introgression but with persistence of pure populations; and (3) complete admixture. Estimates of the frequency, composition and expansion dynamics of hybrids are therefore essential data for assessing the future of a hybrid system and to propose management policies.

Critical cases of human mediated invasion associated with interspecific hybridization appear widespread in the so-called tilapias, a paraphyletic and diverse group of mostly African cichlids. Interspecific hybridization is a pervasive phenomenon in natural population of tilapias (e.g. [14], [15], [16]) that recently turned into a destructive potential due to numerous worldwide introductions of several species for aquaculture purposes. Most of them belong to the genus *Oreochromis* [17], which has been intensively used in aquaculture of tropical and subtropical regions across the world since the 1950s. Anthropogenic translocations of *Oreochromis* within Africa were reported to induce several cases of interspecific hybridization leading to severe threats for the genetic integrity of native local species (e.g. [18], [19], [20], [21]). Moreover, recent studies investigating the transmission of hybrid genomes across generations have demonstrated that hybridization even between highly distantly related tilapia species can lead to classic meiotic processes with diploid Mendelian segregation and maintenance of a stable and recombining hybrid gene pool across generations [22]. Aquaculture performance and yield of many domestic native *Oreochromis* strains bred in Africa became significantly compromised due to inadequate management practices [23], [24]. Consequently, the Nairobi Declaration [25] regarding the management of tilapia aquaculture and biodiversity in Africa underlines the priority to identify and manage wild native stocks of important tilapia species. Thus, conservation genetics of native *Oreochromis* populations are an issue of high concern for the development of new strains as well as for the conservation of African freshwater communities [26].

**Figure 1:**
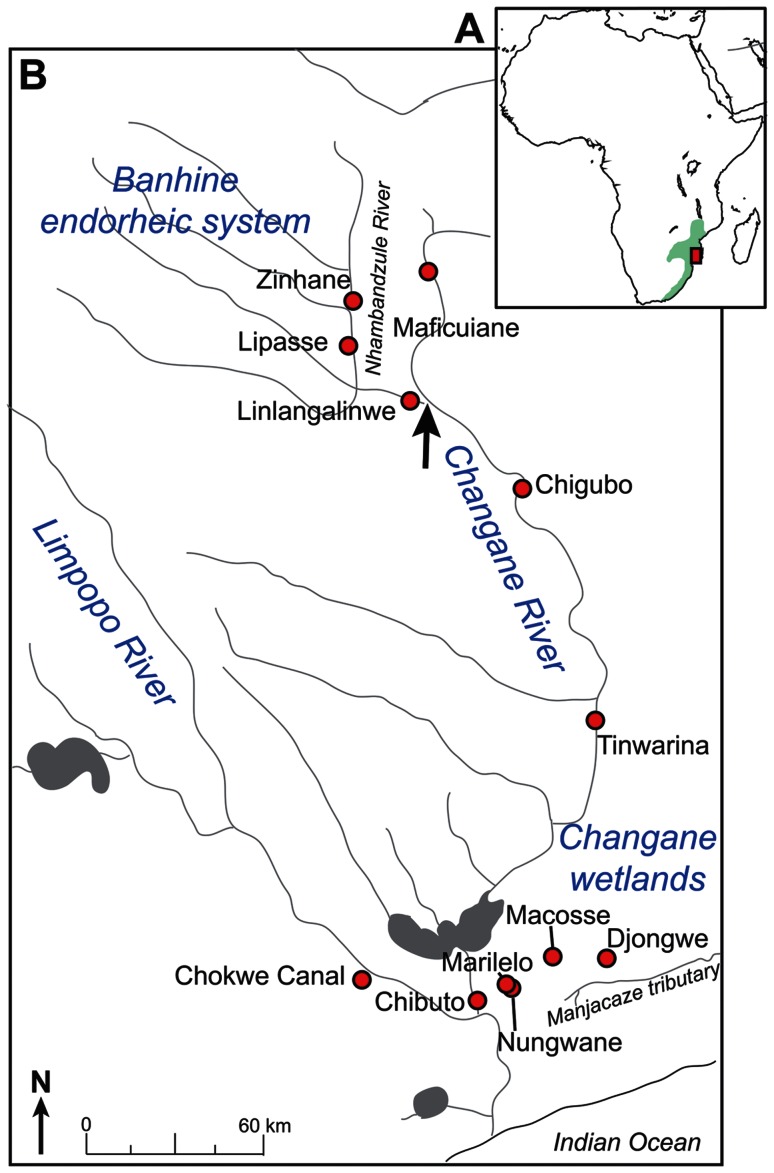
The *O.* *mossambicus* native range and location of the sampling localities (Changane-Lower Limpopo). **A**. Native range of *Oreochromis mossambicus* (green area) and study area (red square). **B**. Locations of the 12 sampling localities in the Changane-Lower Limpopo- system (red circles). The black arrow points the zone of hydrological disconnection of the Banhine endorheic system from the Changane River mainstream.

The Mozambique tilapia, *Oreochromis mossambicus,* is classified as “near threatened” on the IUCN Red List because of its hybridization with the widely introduced *Oreochromis niloticus* [27], native from the nilo-sudanic region. *O. mossambicus* is a well-recognized aquaculture species with a recent human-induced worldwide distribution [28], [29], [30]. It was the first tilapia species spread at a global scale for aquaculture purposes. Its native range (Figure 1A) comprises several drainages of south-eastern Africa from the Eastern Cape (South Africa) in the south to parts of the Zambezi basin in Mozambique in the northern part of its range. It includes the Limpopo basin and several coastal rivers [31]. A large proportion of the *O. mossambicus* geographic distribution lies within the Limpopo River system (South Africa, Botswana and Mozambique).

### Invasion history of the Limpopo Drainage

The Limpopo River system provided the most extensive evidence for the spread of *O. niloticus* and its hybridization with native *O. mossambicus* [19], [32], [33], [34]. *O. niloticus* was probably introduced in the Upper Limpopo system (Zimbabwe) in the early 1990s from a population previously established in Lake Kariba. Several dams in the Upper Limpopo system were stocked with *O. niloticus*, in some cases with several tens of thousands of specimens [35]. Its first record in the Upper Limpopo (South Africa) is from 1996 [33]. Subsequent collections (1998) combined with allozyme analyses revealed the presence of interspecific hybrids [19] which was later confirmed by microsatellite genotyping and mitochondrial DNA (mtDNA) sequences by D′Amato *et al*. [32]. A second documented *O. niloticus* inoculation occurred as the consequence of major floods in the year 2000 leading to the escape of *O. niloticus* specimens from a fish farm located in the Lower Limpopo River [36].

It is noteworthy that *O. niloticus* is not the first alien *Oreochromis* species having spread in the Limpopo System. In 1973, *Oreochromis andersonii* specimens originating from the Okavango River are known to have been released in the Upper Limpopo System (Bostwana) [37]. Its genetic persistence has been confirmed by *O. andersonii* haplotypes recovered in the Upper Limpopo [32].

### Objectives of the study

All previous genetic studies mainly concerned populations sampled in the Upper Limpopo close to well-known zones of introductions [19], [32], [33] with the addition of some minor samples in the Olifants' River (South Africa) [32], thus providing an insightful but incomplete picture of the genetic pattern of the whole drainage. Unfortunately, there are no genetic data for the *Oreochromis* populations of the lower reaches of the Limpopo although this zone is of crucial concern for the conservation of *O. mossambicus* because it potentially shelters genetically pure populations. Moreover, there are no fine-grained genetic data describing the autochthonous structure and diversity of pure *O. mossambicus* populations at a local scale.

The Changane drainage, located in the Gaza Province, Mozambique, is the main tributary of the Lower Limpopo and an intermittent dry land river. It represents the largest and least disturbed wetland of the Limpopo system [38]. Most of the year and especially during the dry season, the Changane River mainstream is reduced to a succession of disconnected ponds with often extreme eutrophic and/or saline conditions, the latter related to soil factors (M. Losseau, personal data). The upper reaches of the Changane River are connected to a seasonally endorheic basin only linked to the rest of the system during rare (i.e. decennial) but major flood events. During the dry period, peculiar geological conditions (see [39]) favor the formation of highly saline swamps in the central part of the system, exposing the freshwater fauna to extreme ecological conditions (total dissolved solids sometime reaching approx. 25 g/L; M. Losseau, unpublished data). The main river channels are fringed by permanent lakes, some isolated from the mainstream, and collectively represent a substantial area [38]. This peculiar hydrological situation therefore provides a fragmented and ecologically heterogeneous system, and constitutes an opportunity to shed light on the factors determining the spread of invasive species and possibly of associated hybrids genomes in the Limpopo system as well as to identify potential *O. mossambicus* populations of high conservation value.

The general objective of the present study is to assess the invasion of alien *Oreochromis* sp. in the Changane-Lower Limpopo *O. mossambicus* metapopulation as a model system, and to characterize the spread of alien and hybrid genomes across geographical and ecological barriers. We genotyped 376 specimens from 12 populations for 423 nuclear AFLP markers and sequenced a subset of 176 specimens for a fast evolving mitochondrial DNA locus in order (1) to estimate the autochthonous diversity and population structure among genetically pure *O. mossambicus* populations with the aim to provide a first baseline for the conservation genetics of this endangered species, (2) to quantify and describe genetic variation of the invasive populations, and investigate the main factors likely to influence their spread and genetic introgression with the native *O. mossambicus*, (3) to identify *O. mossambicus* populations not affected by hybridization.

## Results

### Distribution of mtDNA haplotypes

**Figure 2:**
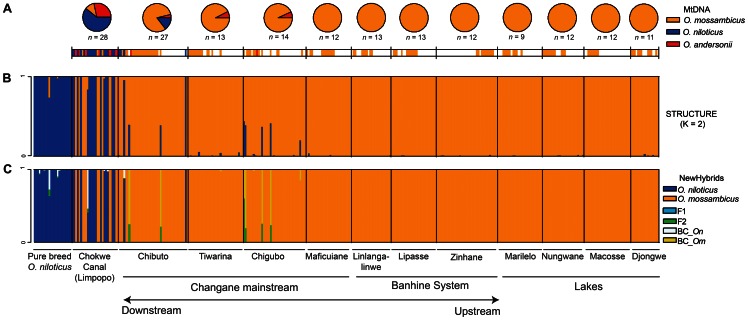
Distribution of mitochondrial haplotypes and AFLP genotypes in the Changane-Lower Limpopo system. **A**. Pie charts of haplotype per species and individual correspondence of the haplotypes with the rest of the figure. **B**. structure barplot for *K* = 2 showing the assignment values of individuals from the 13 localities sampled in the Changane-Lower Limpopo- system. The first group is reference *O. niloticus* samples. **C**. Same plot obtained with newhybrid with two possible parental and four hybrid categories. Geographic locations are described in Figure 1.

**Figure 3:**
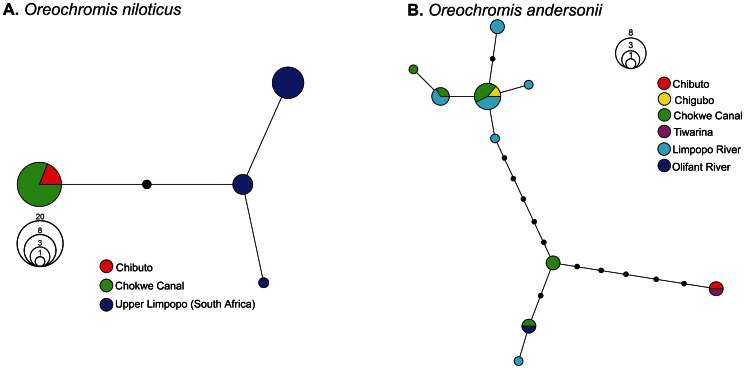
Networks of the two alien species haplotypes recovered in the Changane-Limpopo System. **A**. *Oreochromis niloticus* and **B**. *Oreochromis andersonii*. Black circles represent unsampled haplotypes. Networks included haplotypes sampled in the Limpopo basin and previously published on GenBank. Geographic locations are described in Figure 1.

MtDNA analysis revealed the presence of haplotypes from three *Oreochromis* species in the Limpopo-Changane system (Figure 2A). *O. mossambicus, O. niloticus* and *O. andersonii* haplotypes (Clusters 2, 5 and 6 respectively in ref. [32]) co-occur in the Limpopo River (Chokwe) and in the Lower Changane River close to the connection with the Limpopo (Chibuto). *O. mossambicus* haplotypes are the most frequent across localities (range: 81–100%) except in the Limpopo River where *O. niloticus* haplotypes are dominant (61%). *O. andersonii* haplotypes were recovered in the Limpopo and in the lower and middle reaches of the Changane River and are absent elsewhere. Six distinct haplotypes were recovered in the *O. andersonii* sample (11 individuals; GenBank: JQ907508–JQ907513) while only a single haplotype was found for *O. niloticus* despite the highest number of sampled mtDNA specimens for this species (21 individuals; GenBank: JQ907514). This haplotype differs from the three *O. niloticus* haplotypes previously recovered in the Limpopo (haplotypes C5–6 and C5–2 [a name encompassing two haplotypes] in ref. [32]) (Figure 3A). The set of *O. andersonii* haplotypes of the Chokwe Canal exhibits a high diversity falling within in the range of values of native *O. mossambicus* populations (Table 1). Three of the *O. andersonii* haplotypes were previously recovered in the Limpopo Drainage (haplotypes C6b-1, 6c-2, 6c-3 in ref. [32]) and three others are new (Figure 3B).

**Table 1:** Sampled localities and summary statistics of genetic diversity.

		AFLP			MtDNA						
Locality	Coordinates	N	He	SD-He	Species	N	Nhap	Hd	SD-Hd	π	SD-π
Djongwe	S24°31′22.2′′ E33°56′29.4′′	17	0.0607	0.0055	*O. mossambicus*	11	6	0.7273	0.1444	0.0128	0.0035
Macosse	S24°30′37.3′′ E33°45′10.2"	27	0.0408	0.0043	*O. mossambicus*	12	3	0.7121	0.0691	0.0092	0.0025
Nungwane	S24°37′25.9′′ E33°35′26.6′′	25	0.0446	0.0045	*O. mossambicus*	12	6	0.8788	0.0595	0.0107	0.0029
Marilelo	S24°37′33.4" E33°35′16.6"	27	0.0427	0.0042	*O. mossambicus*	9	5	0.8611	0.0872	0.0104	0.0030
Zinhane	S22°20′19.3" E33°04′04.3"	37	0.0460	0.0044	*O. mossambicus*	12	6	0.8485	0.0744	0.0085	0.0023
Lipasse	S22°28′18.4′′ E33 02′30.0′′	28	0.0412	0.0044	*O. mossambicus*	13	4	0.7179	0.0888	0.0083	0.0022
Linlangalinwe	S22°39′06.1′′ E33°17′21.2′′	24	0.0448	0.0045	*O. mossambicus*	13	8	0.8590	0.0886	0.0077	0.0020
Maficuinae	S22°13′02.6" E33°19′33.4"	27	0.0363	0.0040	*O. mossambicus*	12	4	0.7121	0.1053	0.0065	0.0017
Chigubo	S22°56′49.0" E33°40′39.0"	38	0.0455	0.0043	*O. mossambicus*	13	6	0.8333	0.0815	0.0075	0.0020
					*O. andersonii*	1	1	__	__	__	__
Tiwarina	S23°43′27.0" E33°54′43.7"	33	0.0434	0.0046	*O. mossambicus*	12	4	0.5606	0.1540	0.0051	0.0014
					*O. andersonii*	1	1	__	__	__	__
Chibuto	S24°40′25.6" E33°30′13.1"	42	0.0457	0.0045	*O. mossambicus*	23	9	0.8696	0.0407	0.0190	0.0046
		_	_	_	*O. niloticus*	4	1	0	0	0	0
Chokwe Canal	S24°38′07.0′′ E33°04′37.9′′	28	0.0599	0.0052	*O. mossambicus*	3	3	1.0000	0.2722	0.0106	0.0059
		_	_	_	*O. niloticus*	17	1	0	0	0	0
		_	_	_	*O. andersonii*	8	5	0.8571	0.1083	0.0116	0.0035
Cirad	__	23	0.0414	0.0043	*O. niloticus*	__	__	__	__	__	__

He: Gene diversity (AFLP);

Nhap: number of haplotypes; Hd: haplotype diversity; π: nucleotidic diversity.

SD: standard deviation.

**Figure 4:**
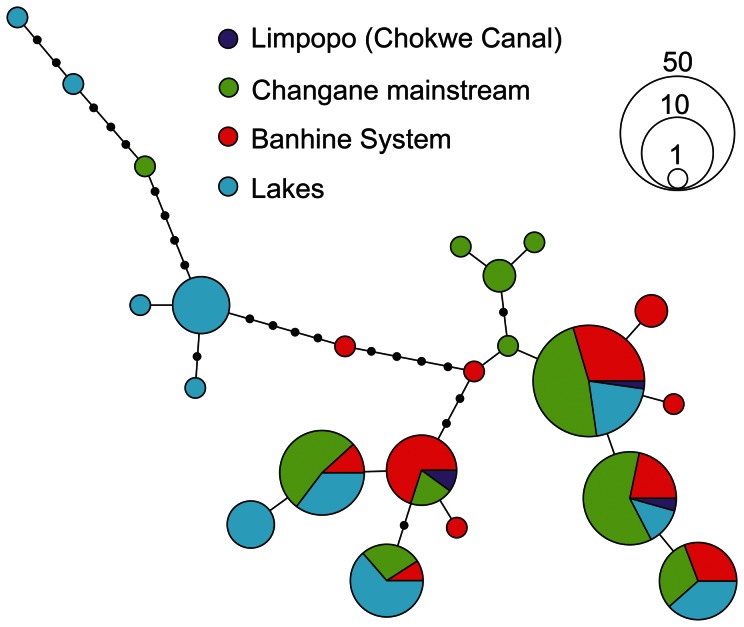
Network of *Oreochromis mossambicus* haplotypes sampled within the Changane-Lower Limpopo system. Black circles represent unsampled haplotypes. Geographic locations are described in Figure 1.

Twenty-two *O. mossambicus* haplotypes were recovered (144 individuals; GenBank: JQ907486–JQ907507). AMOVA indicated a significant differentiation between lacustrine and riverine *O. mossambicus* populations (variance explained = 9.24%; *Φ*_ct_ = 0.13; *P*<0.001) and a significant population divergence between localities (variance explained = 28.44%; *Φ*_st_ *= *0.39; *P*<0.001). The *O. mossambicus* haplotype network (Figure 4) illustrates this pattern with only six out of the 22 haplotypes found both in lakes and the river. Djongwe Lake appears as a notable example since most of the sequenced individuals (10/11) bear haplotypes not recovered elsewhere (Table S1).

### Patterns of hybridization

The analysis of the whole AFLP dataset (deposited in Dryad: doi:10.5061/dryad.k0fs1) using structure indicated that the most likely number of clusters is *K* = 2 according to the Δ*K* criteria (Figure S1). The reference *O. niloticus* individuals and part of the samples from Chokwe Canal and Chigubo are clearly assigned to the first cluster (Figure 2B). Individuals of the second cluster (i.e. *O. mossambicus* genotypes) dominate the samples from the upper and middle reaches of the Changane River and the lakes. Unambiguous evidence for admixed nuclear genotypes was only identified at Chibuto and Chigubo albeit at low frequencies (2/42 individuals [5%] and 5/38 individuals [13%] respectively). The newhybrids analysis indicates a very similar pattern as the one obtained with structure (Figure 2C) and confirms that the previously identified admixed genotypes are *O. mossambicus* backcrosses or later generation *O. mossambicus*-dominant hybrids except for one individual (Chokwe Canal) which is likely to be an *O. niloticus*-dominated hybrid genotype. To summarize, localities likely hosting allochthonous species and hybrid individuals are located in the Limpopo (Chokwe Canal) and in the lower and middle reaches of the Changane sub-drainage (Chibuto, Chigubo). Not surprisingly the highest values of genetic diversity were found in these four samples (Table 1). A second structure analysis performed without the individuals bearing an *O. niloticus* component (i.e. removing admixed genotypes detected in the first clustering analysis to investigate hypothetical hybridization patterns with a third species, *O. andersonii*, see Methods) provides only weak support for a congruent multi-cluster pattern in the data and no support for structured interspecific admixture in the nuclear genome (Figure S2).

Individual comparisons of AFLP based assignments and mtDNA (Figure 2A-C) reveal three individuals exhibiting an *O. mossambicus* nuclear genotype with an *O. niloticus* mitochondrial haplotype. All three occur in the Limpopo (Chokwe Canal, *N = *2) and in the Lower Changane (Chibuto, *N = *1). structure results obtained with *K = *3 indicate that individuals bearing an *O. andersonii* haplotype do not tend to be more likely assigned to the third –hypothetically *O. andersonii*– cluster than specimens with an *O. mossambicus* haplotype (One-sided Wilcoxon rank-sum test: *W* = 613, *P* = 0.462).

### Population genetic structure (AFLPs) within *O. mossambicus*

Population genetic structure within *O. mossambicus* was evaluated including the eight localities showing no trace of alien genotypes. Pairwise *F*_ST_ calculations (Table 2) reveal no differentiation between the four riverine localities (all *F*_ST* = *_0.0000). Of the lakes investigated, Macosse is also not significantly differentiated from the rest of the Changane drainage (all *F*_ST_ ≤ 0.0011). The three other lacustrine localities generally exhibit strong levels of differentiation with all or part of the system (Table 2). Especially, Djongwe Lake exhibits strong levels of differentiation with all other localities except with the lakes Macosse and Nungwane. The results of AMOVA show that variation in the AFLP dataset is weakly but significantly structured according to the lacustrine-riverine distinction (variance explained = 1.32%, *Φ*_ct* = *_0.014, *P = *0.029. The between locality level also explained a low proportion of the variance (variance explained = 3.62%, *Φ*_st* = *_0.025, *P = *0.047).

**Table 2:** Pairwise AFLP *F*_st_ comparisons within the *O. mossambicus* populations preserved from hybridization with alien *Oreochromis* species.

	Maficuiane	Linlangalinwe	Lipasse	Zinhane	Macosse	Marilelo	Nungwane	Djongwe
Maficuiane								
Linlangalinwe	0.0000							
Lipasse	0.0000	0.0000						
Zinhane	0.0000	0.0000	0.0000					
Macosse	0.0000	0.0000	0.0000	0.0010				
Marilelo	0.0019	0.0000	0.0014	0.0047**	0.0011			
Nungwane	0.0046**	0.0000	0.0043**	0.0060**	0.0005	0.0000		
Djonwge	0.0072**	0.0030*	0.0037*	0.0070**	0.0011	0.0031*	0.0000	

Values come from aflp-surv.

* Significant at *P*<0.05 - ** Significant at *P*<0.01.

structure analyses, with and without the locprior option, do not detect differentiation between the *O. mossambicus* populations unaffected by introgression. The most likely number of clusters is *K* = 2 (with locprior) and *K* = 4 or 6 (without locprior) (Figure S3A), with no likelihood gain relative to *K = *1 when using the locprior model. This strongly suggests the absence of clustering in the data, which is further supported by the distribution of the posterior probabilities of individual assignments (*Q*) at *K = *2 to 6 revealing no apparent patterns of clustering for both models (Figure S3B).

## Discussion

Our results show that interspecific hybridization in the Limpopo system leads to occasional introgressions, except in the upper reaches of the Changane River and investigated surrounding lakes where many pure *O. mossambicus* populations persist. This situation fits with scenario #2 defined by Allendorf *et al.* [4]. The presence of hybrids in both the Lower Limpopo and in the lower and middle reaches of the Changane River is in accordance with previously published genetic results for the Upper Limpopo [19], [32], [33]. Our study provides a more comprehensive view of this study system for invasion-mediated hybridization and its dynamics as evidenced by heterogeneous patterns of genetic admixture between localities. With regards to expectations from previous genetic investigations [19], [32], [33], the most striking aspect of our results is the low occurrence of hybrids in the Lower Limpopo, in spite of the strong exposure of this system to allochthonous species evidently able of hybridization (e.g. [40], [41]). This may be related to several factors such as date and patterns of alien *Oreochromis* sp. introduction events, distance from the sites of introduction and local ecological conditions.

### Patterns of hybrid expansion

MtDNA indicates at least two events of alien mtDNA lineages dispersal in the studied system: *O. andersonii* and *O. niloticus*. The first one involved *O. andersonii*, a species released in 1973 approximately 600 km upstream to the Changane River in the Upper Limpopo drainage [37]. Therefore, the *O. andersonii* haplotypes expansion has likely progressed downstream in the Limpopo River and then again upstream up to the middle reaches of the Changane River (Chigubo). The second mtDNA dispersal event has involved *O. niloticus* mtDNA introgression into the *O. mossambicus* gene pool and is evidenced by few individuals in the Limpopo as well as in lower Changane. Hybridization events leading to cytonuclear discordance are not rare in related cichlid species [15], [16] and can occur over contemporary time-scales in an invasion context [20], [42]. Several factors are likely to lead to cytonuclear discordance in tilapias such as unidirectional hybridization or unbalanced sex-ratio of hybrids generations (see [15]). The peculiarity of the Limpopo system is that *O. mossambicus* experienced two recent successive events of partial (and possibly ongoing) mtDNA introgression. *O. mossambicus* x *O. niloticus* successive backcrosses involving female *O. niloticus* are reported from experimental conditions [41] and could have led to the observed pattern in the wild. The fact that mtDNA introgression also occurred from *O. andersonii* to *O. mossambicus* suggests some similarities in the processes of expansion and introgression for both alien species. Unbalanced sex-ratios of interspecific hybrid progeny, a well-documented pattern in tilapias (e.g., [43], [44]), can be hypothesized as a potential common underlying process.

*O. niloticus* haplotypes previously recovered in the Upper Limpopo by D′Amato *et al.* [32] did apparently not reach the lower part of the system where a different haplotype is found. Interestingly, this Upper *vs*. Lower Limpopo geographic segregation of *O. niloticus* haplotypes possibly reflects the two reported introduction events which respectively occurred in the Upper [35] and in the Lower [36] Limpopo. MtDNA therefore probably mirrors historical and geographical patterns of *O. niloticus* introduction in the Limpopo system. The *O. niloticus* mtDNA haplotype, although found at high density close to one of its putative zones of introduction, is less widespread than *O. andersonii* haplotypes. At least three factors constraining the spread of *O. niloticus* relative to *O. andersonii* may have contributed to this differential pattern.

First, the *O. andersonii* introduction likely precedes by at least 15 years the two recognized releases of *O. niloticus* [34], [36], [37]. As a consequence, the rarity of *O. niloticus* in the system may simply correlate with the little time spent since introduction.

Second, a much higher mtDNA diversity was observed in *O. andersonii* indicating that this species did not experience a genetic bottleneck as severe as *O. niloticus*. This agrees with the fact that *O. andersonii* was introduced from a population directly originating from its native range (Okavango Drainage) [37] and is thus supposed to be genetically more diverse than the already translocated invasive propagules (Upper Limpopo) or the aquaculture strain (Lower Limpopo) from which *O. niloticus* was established [35], [36]. MtDNA diversity therefore suggests a higher propagule pressure for *O. andersonii* compared to *O. niloticus*, which would be in accordance with the relative remoteness of the native ranges of the two alien species (see e.g. [32]). However, a single introduction is documented for *O. andersonii* only while at least two independent introductions occurred for *O. niloticus* [35], [36], [37], as supported here by mtDNA. A high propagule pressure coupled with genetic admixture between invasive lineages coming into contact is often suggested as a factor enhancing invasive and adaptive potential through hybrid vigor [8], [9], [45], [46], [47]. For example, this factor likely favored the invasion success of several poorly diverse but interbreeding rainbow trout aquaculture sources [48]. However, the distribution of *O. niloticus* haplotypes suggests that this species may not have benefitted from admixture of its two low diversity sources (Upper and Lower Limpopo sources). Thus, the much higher genetic diversity of *O. andersonii* –as estimated from mtDNA– may have favored its broader expansion in the drainage relatively to *O. niloticus*.

Third and last, *O. andersonii* is phylogenetically closer to *O. mossambicus* than to *O. niloticus* [49]. Thus, genomic incompatibilities with *O. mossambicus* are expected to be fewer with *O. andersonii* than with *O. niloticus* leading to weaker intrinsic barriers to introgression and more likely spread of genetic components of the first species. In summary, time since introduction, patterns of genetic diversity and genetic incompatibilities between alien species and *O. mossambicus* could explain the broader expansion of *O. andersonii* haplotypes in the system. Time since introduction can be considered as a baseline explanation, but differential genetic introgression of *O. andersonii* was potentially accelerated by the other two factors (i.e. phylogenetic distance to *O. mossambicus* and genetic diversity).

A survey of recent invasion-mediated hybridization in *Oreochromis* suggests that rapid replacement and even local extinction of the native or resident species can occur. For example, introduction of *O. niloticus* in the previously established *Oreochromis macrochir* population in a Madagascan lake has led to the complete replacement of *O. macrochir* with *O. niloticus* after only ten years [50]. Similarily, in a dam within the Limpopo drainage, van der Waal [35] reports the replacement of *O. mossambicus* by *O. niloticus* in less than ten years. In a second dam, Weyl [51] documents the invasion of *O. niloticus* in less than one year, but with no evidence for the total replacement of *O. mossambicus* over this short time scale. Less sudden or only a partial genetic replacement can also occur, as exemplified by the partial (27%, *N* = 30) introgression of introduced *Oreochromis leucosticus* mtDNA into a native *O. niloticus* gene pool of the Lake Baringo, Kenya [20]. In the upper part of the Limpopo System, previous studies indicate strong inter-annual variation in the frequency of introduced species and hybrids based on allozymic data [19], [33]. Possibly four to eight years after the first probable *O. niloticus* introduction in the early 1990′s [35], Moralee *et al.* [19] reported 86% of *O. niloticus* or hybrids and 14% of *O. mossambicus* (*N = *257). Using a similar allozyme based approach for fish sampled in 2002 and 2006, van der Bank and Deacon [33] only identified 25% of alien species or hybrids in 2002 (*N = *63) and 35% in 2006 (*N = *103) suggesting that the frequency of preserved *O. mossambicus* genotypes can drastically vary with time in a riverine system.

Our results also show that hybrid frequency and invasion patterns strongly vary spatially suggesting a pattern of progressive and only localized replacement. Furthermore, *O. niloticus* expansion and *O. mossambicus* local extinction in the Lower Limpopo are not as dramatic as previously reported for invaded lakes [35], [51] and for some sections of the Upper Limpopo [19], [33], [34]. Riverine systems such as the Changane River are exposed to annual floods and droughts that may drastically alter patterns of genetic connectivity between cichlid populations over short time scales [52]. Pure *O. mossambicus* specimens remain dominant in the most isolated and remote parts of the system (here the upstream section and the surrounding lakes). Noteworthy, our population genetic data agree with a recent qualitative ecological study of *O. niloticus* invasion risk in the Limpopo [53] indicating that headwater regions are the least threatened by the *O. niloticus* invasion. Thus, temporarily isolated and still unintrogressed headwater *O. mossambicus* populations could act as refugia preventing the total replacement by alien species and hybrids of the native Changane populations.

A mosaic of extreme environmental conditions brought together is found along the Changane River system, from highly saline or brackish swamps in the lower and middle reaches to eutrophic freshwater swamps in the head river sections (M. Losseau, unpublished data). The heterogeneous pattern of admixed genotypes distribution in the Lower and Middle Changane River may partly result from highly variable ecological conditions possibly acting as barriers to the expansion of potentially less resistant alien or admixed genotypes. Extreme habitats characterized by long periods of extreme eutrophic conditions, such as swamps in the river bed, are expected to challenge the establishment and spread of alien species that would be less favored under hypoxia and low water temperature [53] than the supposedly locally adapted *O. mossambicus* populations. Given the low prevalence of hybridization, competition for food between *O. niloticus* and locally preadapted *O. mossambicus* should also be considered as a factor depriving fitness of *O. niloticus*, as a trophic niche overlap of the two species was documented in the Limpopo [54]. Disentangling the respective contributions of geographical, ecological and reproductive barriers responsible for the maintenance the genetic integrity of relictual populations now appears as a primary topic for the conservation of *O. mossambicus*. Diachronic genetic surveys before and after major flood events (e.g. [52]) could as well allow estimating the fragility of contemporary genetic structures when faced to temporary cessations of gene flow due to transient geographic and ecological barriers. Extensive genomic scan approaches (see e.g. [55]) performed at a broad geographic scale and including well known functional loci in tilapia (e.g. [56]) would also help to identify the adaptive genetic divergence among several preserved populations occupying contrasted conditions. Such an approach and dataset would be demanding in terms of sampling effort, but represents a next step to evaluate the vulnerability of *O. mossambicus* conservation units and the potential impact of the invasion-mediated hybridization on the dilution and loss of local adaptive variation. The evidence for at least second generation hybrids in this system (although rather rare) and the broad amount of data now available on the *Oreochromis* genome could provide the opportunity for future investigation of both the genomic location and function of putatively non-neutrally introgressing alleles in the biological invasion context. This could be achieved by SNPs genotyping and mapping using a next generation sequencing approach (e.g. RAD sequencing) [57], [58].

### Genetic structure and diversity in native *O. mossambicus*

The preserved *O. mossambicus* populations recovered in the Changane system exhibit a substantial amount of genetic diversity contrasting with the depleted genetic diversity reported for *O. mossambicus* populations that were exported worldwide during the 20^th^ century [59], [60], [61]. Populations from the Changane system, therefore, could represent potential sources for *O. mossambicus* restocking in critically invaded areas (e.g. the Upper and mainstream Limpopo) as well as a diverse autochtonous genetic resource for the development of new local aquaculture strains [26].

Although the clustering approach indicates the relative homogeneity of the preserved *O. mossambicus* gene pool, a significant differentiation between riverine and lacustrine habitats for both nuclear and mitochondrial markers indicates that the *O. mossambicus* populations included in this study could represent at least two distinct conservation units related to their geographical distribution and/or ecological versatility. The null *F*_ST_ values found between the four sampling sites of the headwater region despite the current strong isolation of each swamp has to be considered with regards of the recent history water flow variation. A major flood occurred in 2000, which had connected all swamps hydrologically and hereby allowed homogenization of genetic variation. Sampled localities were disconnected two or three years thereafter as a result of increased drought. Riverine populations therefore have remained permanently isolated over the last five or six years. This time lapse was probably too short to lead to detectable genetic differentiation between these localities. Floods have already been evidenced as a radical homogenizing factor erasing isolation by distance patterns in riverine cichlids [52]. We hypothesize that this is the case in the Changane River system too, with temporal variation in genetic structure due to prolonged drought phases alternated with extensive flood events allowing for amalgamation of intermittently isolated fish populations.

The lacustrine *vs.* riverine differentiation is further supported by the highest *F*_st_ found among comparisons involving three out of four isolated lacustrine sites (Marilelo, Nungwane, Djongwe) *vs.* the four riverine headwater sites (Zinhane, Lipasse, Linlangalinwe, Maficuiane). Macosse Lake, which represents the largest permanent body of water included in this study, exhibits no significant genetic differentiation from the rest of the system. The presence of several shared and frequent haplotypes between lakes and the river could suggest that lacustrine populations result from multiple colonization events. Overall, geographic isolation over geological time scales (i.e. since the last major Pleistocene sea level fluctuation or extreme floods events connecting lakes to rivers), possibly combined with an increased drift effect in populations from small water bodies could have induced the within-drainage differentiation pattern. Interestingly, a recent study of native *O. niloticus* populations also reports significant values of local-scale genetic differentiation associated to the levels of geographic connectivity between populations [62]. The D′Amato *et al.* [32] analyses performed at the scale of the whole *O. mossambicus* native range indicated genetic differentiation among drainages. Focusing on a narrower geographical scale with an intensive sampling, the present study provides a finer picture of the *O. mossambicus* local genetic structure, indicating that the naturally fragmented *O. mossambicus* habitat induced subtle genetic within-drainage differentiation. These are potential conservation units to be managed locally (here, riverine and lacustrine populations).

## Conclusion

Our results provide rather hopeful auspices for the conservation of *O. mossambicus* in the face of introductions of allochthonous *Oreochromis* species in the Limpopo drainage. We provide evidence for only a limited expansion of alien species and their hybrids in the Lower Limpopo despite multiple introductions. This indicates that the spread of hybrids in the system is rather slow probably due to geographical and ecological barriers. The peculiarity of the Limpopo hybrid system is that the endangered *O. mossambicus* underwent two successive waves of interspecific genetic introgression from introduced species. Currently, these two invasive species exhibit remarkably different levels of genetic variation that potentially correlate with their respective invasive abilities. This result encourages further investigations on the role of propagule pressure and genetic diversity in the success of biological invasions involving genetic introgression of locally adapted native species. In addition, we identified two refugial zones that should henceforth receive high priority for the conservation of *O. mossambicus*: the head of the Changane drainage and the lakes surrounding the Lower Changane River. Considering that the four investigated lakes are exclusively populated by native *O. mossambicus* strongly suggests that the surrounding wetland system around the Changane (a considerable water surface [38]) represents an ideal refugial zone for the species. The absence of genetic structure among native riverine *O. mossambicus* populations suggests that major floods may help to homogenize temporarily isolated riverine populations. Furthermore, we also show that hybrids are able to spread over long distances in an upstream direction. Accordingly, it can be expected that, in the long term, the genetic integrity of riverine populations will be threatened by hybrid expansion mediated by future flood events. The genetic integrity of the more isolated lacustrine populations is therefore less precarious over the long term.

Recently, Tweddle and Wise [35] noticed that “There is a strong economic argument to allow the culture of Nile Tilapia in the Limpopo catchment as this has already been invaded”. Our results support that the entire Limpopo system is far from being uniformly invaded and hosts preserved and possibly locally adapted *O. mossambicus* populations that clearly deserve the attention of conservationists, wildlife managers as well as of tilapia aquaculture development plans and genetic improvement programs. Concerning tilapia aquaculture, *O. mossambicus* has been widely used for two important traits, its impressive euryhalinity and its propensity to provide reddish-orange mutants of high commercial value. Therefore, *O. mossambicus* has been systematically used to produce new salinity-resistant commercial strains or/and red tilapia strains [63], [64].

Preventing people and aquaculture companies from stocking *Oreochromis* sp. in these localities and prohibiting the development of allochtonous tilapia aquaculture in the Limpopo region should therefore be considered among the first conservation actions. The Changane *O. mossambicus* populations represent good candidates for the development of *O. mossambicus* aquaculture strains that could be used in the already invaded zones of the Limpopo. As a final remark, we note that this genetic richness for world aquaculture also and primarily represents a significant protein resource for the inhabitants of the Gaza Province, an area comprising the poorest districts of Mozambique [65]. The principal challenge is now to manage and preserve these relictual populations in agreement with the local people, i.e. without compromising their access to this food resource.

## Materials and Methods

### Sampling procedure and DNA isolation

All necessary permits for sampling were obtained from the University Edouardo Mondlane - Faculty of Science (Maputo). A fishing permit was acquired from the Banhine National Park Administration (#0002/2009). At each sampling site local authorities and communities were first contacted and sampling activities always took place with their agreement. The field studies did not involve protected species. Specimens were collected along the Changane drainage and in the Limpopo between 2006 and 2009. A total of 12 sites were sampled, and included the different reaches of the Changane River, surrounding lakes and the Lower Limpopo (Figure 1; Table 1). The selected sites provide landmarks for the progression of alien species and hybrids across the region and represent different ecological conditions and variable degrees of hydrological isolation from the Limpopo mainstream (M. Losseau, unpublished data). Two of the selected sites were supposedly invaded by alien *Oreochromis* sp. and include the Chokwe canals along the main Lower Limpopo drainage and Chibuto close to the confluence of the Changane with the Limpopo. All other sampling sites had different degrees of isolation from the Limpopo and included four endorheic lakes (Nungwane, Marilelo, Macosse, Djongwe) three river pools within the Changane (Tinwarina, Chigubo, Maficuiane) and, finally, three pools within the endorheic headwater system (Linlangalinwe, Lipasse, Zinhane). Tinwarina and Chigubo are permanent water bodies respectively characterized by brackish (up to ca. 7 g/L total dissolved solids) and saline waters (up to ca. 25 g/L total dissolved solids) due to local soil conditions (M. Losseau, unpublished data). The last site (Maficuiane) is an ephemeral pool in the river bed situated about 30 km down the river's origin. The three sampling sites chosen within the headwater endorheic system are all also ephemeral. Zinhane and Lipasse are permanent pools with highly alternating water levels in response to drought; both are located on the Nhambandzule River, which runs from the north into the central wetlands of the Banhine National Park (BNP). Linlangalinwe represents a pool located on the Chefu River, the channel connecting the BNP and the Changane main channel during major flood periods.

In addition, pure-breed *O. niloticus* specimens from a the Bouaké strain (CIRAD unit, Montpellier, France) were included in the analysis as *O. niloticus* reference samples. The Bouaké cultured strain was spread in several regions of Africa [60]. Previous genetic analyses indicated its mixed origin (Volta and Nile drainages) [15] and a high level of nuclear polymorphism [66]. At least two arguments support the idea that the use of this *O. niloticus* strain does not affect the detection of hybrids. First, within the *Oreochromis* radiation *O. niloticus* is a phylogenetically clearly distinct from *O. mossambicus* (and *O. andersonii*) [32], [49], [67], [68]. As a consequence, within species genetic variation (i.e. among *O. niloticus* populations) should have no influence on the following Bayesian approaches and, therefore, can be neglected for the detection of *O. niloticus* hybrids within the Limpopo-Changane system. Second, the AFLP nuclear genetic diversity (*He*, Table 1) of the *O. niloticus* sample (*He* = 0.0414) falls within the estimated Mean ± 1SD range of the among *O. mossambicus* populations variation in diversity (*He* = 0.0446 ± 0.0071). This supports the high polymorphism of the Bouaké strain hosted in Montpellier (in agreement with Bezault *et al.* [66]) and a level of inbreeding similar to the one found in wild *Oreochromis* populations.

Specimens were euthanatized with an overdose of clove oil and a pectoral fin-clip was taken and preserved in 96% ethanol. Total genomic DNA was extracted using Qiagen DNeasy® Tissue Kit according to the manufacturer's protocol and adjusted to a standard concentration of 25 ng/ µL.

### Mitochondrial DNA sequencing

For 176 individuals, we amplified and sequenced a 385 bp fragment of the mtDNA control region based on the protocol of D′Amato *et al.* [32]. PCR conditions were: 5 min at 94°C; then 35 cycles of 94°C for 30 s, 58°C for 40 s, 72°C for 45 s, followed by 72°C for 5 min. Purified PCR fragments were sequenced by the Macrogen® Sequencing Service using a standard procedure.

### AFLP genotyping

We followed a version of the Vos *et al.* 's original protocol [69] as modified according to Herder *et al*. [70]. DNA fingerprints for 376 individuals were generated for six restrictive primer pair combinations (*Eco*RI-ACA/*Mse*I-CAA; *Eco*RI-ACA/*Mse*I-CTT; *Eco*RI-ACT/*Mse*I-CTC; *Eco*RI-ACC/*Mse*I-CTG; *Eco*RI-ACT/*Mse*I-CTG; *Eco*RI-ACT/*Mse*I-CAC). Fragments were separated on an ABI 3130 Genetic Analyzer (PE Applied Biosystem, Foster City, CA, USA) with an internal size standard (ROX 500 XL). The quality of each run was visually inspected. Fragments were scored for each primer combination between 80 and 500 bps using GeneMapper® v.4.0 with a fluorescence detection threshold of 50 units.

Genotyping error rate was estimated using the ratio of mismatches to the total number of replicated markers [71], [72] for a total of 16 replicated samples during the entire procedure (mean: 3.9 replicates per sets; range: 2 – 9 and 62 total replicated samples representing 16.2% of the total sample). The final repeatability was 97.8% for 423 polymorphic loci (range: 56 – 89 loci per primer combinations, Table S2). This high number of dominant markers justifies the choice of an AFLP genotyping approach in a Bayesian assignment context (see above): a recent modeling study [73] has shown that “dominant markers studies can achieve an accuracy similar to that of codominant markers studies if the number of markers used in the former is about 1.7 times larger than in the latter”. In our case, to obtain an assignment accuracy similar to the one expected from our dominant marker set, about 250 co-dominant markers (i.e.∼423/1.7) would have been necessary; an amount of markers rarely observed in, e.g., microsatellite studies.

### Data analyses

#### mtDNA data analyses

MtDNA sequences were aligned using the ClustalW algorithm [74] in BioEdit [75] and corrected manually. *Oreochromis* control region haplotypes already revealed a strong species level taxonomic clustering under parsimony in a previous study [32]. Thus, species assignments of the new haplotypes were obtained using a statistical parsimony haplotype network [76] including already published sequences (Figure S4; Table S3) with the functions *haplotype()* and *haploNet()* of the ‘*pegas’* v0.4-2 package [77] as implemented in R [78] and treating gaps as a character state. Following an identical procedure, detailed within-species haplotype networks were then computed using sequences recovered in the Changane-Limpopo system. Nei's [79] nucleotide diversity and haplotype diversity were computed by locality and species (when a mixture of species haplotypes is recovered for a given locality, see Results), respectively, with the *nuc.div()* function of *‘pegas’* [77] and a custom R routine. A hierarchical analysis of molecular variance (AMOVA) [80] was performed to evaluate levels of differentiation. To test if lacustrine populations represent a distinct genetic group relative to riverine ones, we used a two level AMOVA model: the first level opposes riverine to lacustrine localities and the second one considers variation between localities within the first level. AMOVA was carried out using the *amova()* function of the ‘*pegas*’ package [77] and significance was assessed after 10 000 permutations.

#### AFLP data analyses

Since the dataset includes localities sampled over several years (i.e. Nungwane, Marilelo, Chibuto, Macosse), a preliminary analysis was performed to test for significant chronological differentiation in computing *F*_st_ between years within each sampling site in aflp-surv [81] using the Zhivotovsky [82] 's Bayesian procedure and a uniform prior distribution to estimate allele frequencies from dominant markers. Significance was estimated based on 10 000 permutations. No significant *F*_st_ values were detected (all among years *F*_st_ = 0.0000) and samples of each locality from different years were therefore pooled.

aflp-surv was used to estimate expected nuclear genetic diversity (*He*) per sampling locality using the same procedure. Two different approaches were selected to identify hybrids or pure individuals. The Bayesian clustering method implemented in structure 2.3 [83] takes into account dominant data [84] and was used to test for significant patterns of clustering. Analyses were run ten times for a number of clusters (*K*) ranging from 1 to 13 using a 100000 steps burn-in period followed by 400000 MCMC repetitions. We did not use the clustering model considering sample group information (the locprior option) [85] since we assumed the potential co-occurrence of individuals from several genetic clusters within the same locality. The optimal *K* was determined from the log probability of data given *K* using the Δ*K* criterion [86]. Since three *Oreochromis* species where recovered in the Limpopo drainage from mtDNA [32], we also checked the results provided by structure for *K = *3 in order to detect a geography-related structure or a correlation with *O. andersonii* haplotypes for the individual posterior probability of assignment to the third cluster. Results from structure were compared to those of newhybrids software [87], [88], which computes the posterior probabilities for each individual to be assigned to the following classes: *O. mossambicus*, *O. niloticus*, first generation hybrids (F_1_), second generation hybrids (F_2_) and backcrosses with each parental species (BC*_mossambicus_*, BC*_niloticus_*). This program was run with uniform priors and with 100000 steps burn-in period followed by 300000 iterations to estimate individual posterior probabilities.

As *O. mossambicus* and *O. niloticus* are phylogenetically distinct species, an *O. niloticus* component could hide a consistent amount of genetic variation potentially including a finer scale sub-clustering pattern not detected within the non-*niloticus* cluster. We therefore used a subset of the original dataset from which the individuals previously assigned by structure to the *O. niloticus* cluster (*P*>5%) were removed (i.e. twelve localities, 327 individuals and 316 polymorphic loci in the new final dataset), and ran structure as before.

We then focused on population structure within *O. mossambicus* localities likely unaffected by other species, using a second subset of the original dataset including only populations for which no clues for hybridization were detected in the prior analyses (i.e. eight populations, 212 individuals and 277 polymorphic loci). Genetic differentiation was measured by calculating pairwise *F*_st_ from allelic frequencies computed with the Bayesian method [82] in aflp-surv [81]. The significance of *F*_st_ was determined based on 10000 permutations with Bonferroni corrections for multiple tests (i.e. standardizing the significance threshold by 28, the number of pairwise comparisons). As for mtDNA (see above), we performed an AMOVA on the AFLP dataset. A structure analysis was finally performed using a similar procedure as for the full dataset (see above). Here, following the recommendations of Hubisz *et al*. [85], we used both the new (with locprior) and the original (no locprior) clustering models. structure analyses were carried out at the Bioportal server of the University of Oslo (www.bioportal.uio.no) [89]. The displayed structure *Q* plots correspond to the runs with the most positive log probability of the data for a given *K*.

## Supporting Information

Figure S1 **STRUCTURE analysis of the full AFLP dataset.** Averaged log probability of the data Ln P(*X*|*K*) (upper panel) and the value of the *ΔK* criteria (lower panel) computed according to Evanno *et al*. (2005) for each number of cluster *K*. The *ΔK* plot clearly supports the presence of two clusters in the data.
(TIF)

Figure S2 **STRUCTURE analysis of the AFLP dataset comprising only individuals with no detected** ***O. niloticus*** **component.** **A**. Averaged log probability of the data Ln P(*X*|*K*) (upper panel) and the value of the *ΔK* criteria (lower panel) computed according to Evanno *et al*. (2005) for each number of cluster *K*. **B**. STRUCTURE barplots for *K* = 2 to 7 showing assignment values (*Q*) of individuals with no *O. niloticus* component.
(PDF)

Figure S3 **STRUCTURE analysis of the AFLP dataset only comprising** ***O. mossambicus*** **individuals from the eight localities preserved from genetic introgression.** A. Averaged log probability of the data Ln *P*(*X*|*K*) (upper panels) and the value of the *ΔK* criteria (lower panels) computed according to Evanno et al. (2005) for each number of cluster *K*, with (left) and without (right) the LOCPRIOR option. B. STRUCTURE barplots for *K* = 2 to 6 showing the assignment values (*Q*) of *O. mossambicus* individuals from the eight localities preserved from genetic introgressions, with (left) and without (right) the LOCPRIOR option.
(PDF)

Figure S4 **Haplotype genealogy of the genus** ***Oreochromis*** **based on a 385 bp fragment of the mitochondrial control region.** The size of the circles representing each haplotype is proportional to log(*N* individuals).
(PDF)

Table S1 **Abundance and repartition of haplotypes in the Changane-Lower Limpopo System per locality.**
(PDF)

Table S2 **Number of AFLP loci per primer combination.**
(PDF)

Table S3 **Control region sequences from GenBank used in this study.** Sequences marked with * were included in the haplotypes networks of the figures 3 and 4.
(PDF)

## References

[1] Crispo E, Moore J, Lee-Yaw J, Gray S, Haller B (2011) Broken barriers: Human-induced changes to gene flow and introgression in animals: An examination of the ways in which humans increase genetic exchange among populations and species and the consequences for biodiversity. Bioessays 33: 508–518. 21523794 10.1002/bies.201000154

[2] Rhymer JM, Simberloff D (1996) Extinction by hybridization and introgression. Annual Review of Ecology and Systematics 27: 83–109.

[3] Seehausen O, Takimoto G, Roy D, Jokela J (2008) Speciation reversal and biodiversity dynamics with hybridization in changing environments. Molecular Ecology 17: 30–44. 18034800 10.1111/j.1365-294X.2007.03529.x

[4] Allendorf FW, Leary RF, Spruell P, Wenburg JK (2001) The problems with hybrids: setting conservation guidelines. Trends in Ecology & Evolution 16: 613–622.

[5] Seehausen O (2004) Hybridization and adaptive radiation. Trends in Ecology & Evolution 19: 198–207. 16701254 10.1016/j.tree.2004.01.003

[6] Nolte AW, Tautz D (2010) Understanding the onset of hybrid speciation. Trends in Genetics 26: 54–58. 20044166 10.1016/j.tig.2009.12.001

[7] Prentis PJ, Wilson JRU, Dormontt EE, Richardson DM, Lowe AJ (2008) Adaptive evolution in invasive species. Trends in Plant Science 13: 288–294. 18467157 10.1016/j.tplants.2008.03.004

[8] Ellstrand NC, Schierenbeck KA (2000) Hybridization as a stimulus for the evolution of invasiveness in plants? Proceedings of the National Academy of Sciences 97: 7043–7050. 10.1073/pnas.97.13.7043 PMC34382 10860969

[9] Facon B, Jarne P, Pointier JP, David P (2005) Hybridization and invasiveness in the freshwater snail *Melanoides tuberculata*: hybrid vigour is more important than increase in genetic variance. Journal of Evolutionary Biology 18: 524–535. 15842482 10.1111/j.1420-9101.2005.00887.x

[10] Nolte AW, Freyhof J, Stemshorn KC, Tautz D (2005) An invasive lineage of sculpins, *Cottus* sp (Pisces, Teleostei) in the Rhine with new habitat adaptations has originated from hybridization between old phylogeographic groups. Proceedings of the Royal Society B-Biological Sciences 272: 2379–2387. 10.1038/rspb.2005.3231 PMC1559961 16243698

[11] Verhoeven KJF, Macel M, Wolfe LM, Biere A (2011) Population admixture, biological invasions and the balance between local adaptation and inbreeding depression. Proceedings of the Royal Society B-Biological Sciences 278: 2–8. 10.1098/rspb.2010.1272 PMC2992731 20685700

[12] Sievers C, Willing E-M, Hoffmann M, Dreyer C, Ramnarine I, et al (2012) Reasons for the invasive success of a guppy (*Poecilia reticulata*) population in Trinidad. PLoS ONE 7: e38404. 22693621 10.1371/journal.pone.0038404 PMC3365015

[13] Muhlfeld CC, Kalinowski ST, McMahon TE, Taper ML, Painter S, et al (2009) Hybridization rapidly reduces fitness of a native trout in the wild. Biology Letters 5: 328–331. 19324629 10.1098/rsbl.2009.0033 PMC2679930

[14] Agnèse J-F, Adèpo-Gourène B, Pouyaud L (1998) Natural hybridisation in tilapias. In: Orstom, editor. Genetics and Aquaculture in Africa.

[15] Rognon X, Guyomard R (2003) Large extent of mitochondrial DNA transfer from *Oreochromis aureus* to *O. niloticus* in West Africa. Molecular Ecology 12: 435–445. 12535094 10.1046/j.1365-294x.2003.01739.x

[16] Schliewen UK, Klee B (2004) Reticulate sympatric speciation in Cameroonian crater lake cichlids. Frontiers in Zoology 1: 5. 15679917 10.1186/1742-9994-1-5 PMC544937

[17] Canonico GC, Arthington A, McCrary JK, Thieme ML (2005) The effects of introduced tilapias on native biodiversity. Aquatic Conservation-Marine and Freshwater Ecosystems 15: 463–483.

[18] Angienda P, Lee H, Elmer K, Abila R, Waindi E, et al (2011) Genetic structure and gene flow in an endangered native tilapia fish (*Oreochromis esculentus*) compared to invasive Nile tilapia (*Oreochromis niloticus*) in Yala swamp, East Africa. Conservation Genetics 12: 243–255.

[19] Moralee RD, van der Bank FH, van der Waal BCW (2000) Biochemical genetic markers to identify hybrids between the endemic *Oreochromis mossambicus* and the alien species, *O. niloticus* (Pisces: Cichlidae). Water SA 26: 263–268.

[20] Nyingi DW, Agnèse JF (2007) Recent introgressive hybridization revealed by exclusive mtDNA transfer from *Oreochromis leucostictu*s (Trewavas, 1933) to *Oreochromis niloticus* (Linnaeus, 1758) in Lake Baringo, Kenya. Journal of Fish Biology 70: 148–154.

[21] Gregg RE, Howard JH, Shonhiwa F (1998) Introgressive hybridization of tilapias in Zimbabwe. Journal of Fish Biology 52: 1–10.

[22] Bezault E, Rognon X, Clota F, Gharbi K, Baroiller J-F, et al (2012) Analysis of the Meiotic Segregation in Intergeneric Hybrids of Tilapias. International Journal of Evolutionary Biology 2012: 10. 10.1155/2012/817562 PMC3385623 22779030

[23] Brummett RE (2008) Genetic quality of cultered tilapia stocks in Africa. World Aquaculture 39: 46–72.

[24] Brummett RE, Ponzoni RW (2009) Concepts, alternatives, and environmental considerations in the development and use of improved strains of tilapia in African aquaculture. Reviews in Fisheries Science 17: 70–77.

[25] WorldFish Center (2002) Nairobi Declaration: Conservation of Aquatic Biodiversity and Use of Genetically Improved and Alien Species for Aquaculture in Africa. Penang: The WorldFish Center.

[26] Lind CE, Brummett RE, Ponzoni RW (2012) Exploitation and conservation of fish genetic resources in Africa: issues and priorities for aquaculture development and research. Reviews in Aquaculture 4: 125–141.

[27] Cambray J, Swartz E (2007) *Oreochromis mossambicus* IUCN Red List of Threatened Species. Available: www.iucnredlist.org.Accessed July 2012.

[28] LoweS,Browne M,BoudjelasS,de PoorterM.100 of the world's worst invasive alien species database; 2000; Aliens newsletter, Auckland.

[29] Costa-Pierce B (2003) Rapid evolution of an established feral tilapia (*Oreochromis* spp.): the need to incorporate invasion science into regulatory structures. Biological Invasions 5: 71–84.

[30] Firmat C, Schliewen UK, Losseau M, Alibert P (2012) Body shape differentiation at global and local geographic scales in the invasive cichlid *Oreochromis mossambicus* . Biological Journal of the Linnean Society 105: 369–381.

[31] Skelton PH (2001) A complete guide to the freshwater fishes of Southern Africa. Cape Town: Struik Publishers.

[32] D′Amato ME, Esterhuyse MM, van der Waal BCW, Brink D, Volckaert FAM (2007) Hybridization and phylogeography of the Mozambique tilapia *Oreochromis mossambicus* in southern Africa evidenced by mitochondrial and microsatellite DNA genotyping. Conservation Genetics 8: 475–488.

[33] van der Bank FH, Deacon A (2007) Increased backcrossing has reduced the usefulness of morphological and allozyme data for identifying *Oreochromis niloticus*, *O. mossambicus* (Teleostei: Cichlidae) and their hybrids in the Pafuri reach of the Luvuvhu River in the Kruger National Park, South Africa. Afr J Aquat Sc 32: 193–196.

[34] van der Waal B, Bills R (2000) *Oreochromis niloticus* (Teleostei: Cichlidae) now in the Limpopo River System. South African Journal of Sciences 96: 47–48.

[35] Tweddle D, Wise R (2007) Nile Tilapia (*Oreochromis niloticus*). The economic impact and appropriate management of selected invasive alien species on the African continent, Appendix 3. In: Wise R, van Wilgen B, Hill M, Schulthess F, Tweddle D et al.., editors.Final Report prepared for the Global Invasive Species Programme .

[36] Schneider MF (2003) Occurence of commercial exotic species in the Limpopo River since the floods in 2000 (in Portuguese). Boletim de Investigação Florestal December 2003: 31–36.

[37] de Moor IJ, Bruton MN (1978) Atlas of alien and translocated indigenous aquatic animals in southern Africa. A report of the Committee for Nature Conservation Research National Programme for Ecosystem Research. Port Elizabeth, South Africa.

[38] Hugues RH, Hugues JS (1992) A directory of African wetlands. Cambridge: IUCN, UNEP, WCMC.820 p.

[39] FAO Subregional Office for Southern and East Africa Harare (2004) Drought impact mitigation and prevention in the Limpopo River Basin. In: Food and Agriculture Organization of the United Nations , editor.

[40] Eknath AE, Hulata G (2009) Use and exchange of genetic resources of Nile tilapia (*Oreochromis niloticus*). Reviews in Aquaculture 1: 197–213.

[41] Mateo D, Aguilar R, Campos W, Katalbas M, Severa F, et al. (2004) Salinity tolerance of *Oreochromis niloticus* and *O. mossambicus* F1 hybrids and their successive backcrosses. In: R. Bolivar R, Mair G, Fitzsimmons K, editors. Proceeding of the Sixth International Symposium on Tilapia Aquaculture Manila, Philippines: Bureau of Fisheries and Aquatic Resources. pp. 426–438.

[42] Darling JA (2011) Interspecific hybridization and mitochondrial introgression in invasive *Carcinus* shore crabs. PLoS ONE 6: e17828. 21423759 10.1371/journal.pone.0017828 PMC3056785

[43] Desprez D, Briand C, Hoareau MC, Mélard C, Bosc P, et al (2006) Study of sex ratio in progeny of a complex *Oreochromis* hybrid, the Florida red tilapia. Aquaculture 251: 231–237.

[44] Mair GC, Scott AG, Penman DJ, Skibinski DOF, Beardmore JA (1991) Sex determination in the genus *Oreochromis*. 2. Sex reversal, hybridization, gynogenesis and triploidy in *Oreochromis aureus* Steindachner. Theoretical and Applied Genetics 82: 153–160. 24213059 10.1007/BF00226206

[45] Lockwood JL, Cassey P, Blackburn T (2005) The role of propagule pressure in explaining species invasions. Trends in Ecology & Evolution 20: 223–228. 16701373 10.1016/j.tree.2005.02.004

[46] Facon B, Pointier JP, Jarne P, Sarda V, David P (2008) High genetic variance in life-history strategies within invasive populations by way of multiple introductions. Current Biology 18: 363–367. 18334202 10.1016/j.cub.2008.01.063

[47] Simberloff D (2009) The role of propagule pressure in biological invasions. Annual Review of Ecology Evolution and Systematics 40: 81–102.

[48] Consuegra S, Phillips N, Gajardo G, de Leaniz CG (2011) Winning the invasion roulette: escapes from fish farms increase admixture and facilitate establishment of non-native rainbow trout. Evolutionary Applications 4: 660–671. 25568013 10.1111/j.1752-4571.2011.00189.x PMC3352532

[49] Trewavas E (1983) Tilapiine fishes of the genera *Sarotherodon*, *Oreochromis* and *Danakilia*. Ithaca New York: Cornell University Press.583 p.

[50] Daget J, Moreau J (1981) Hybridation introgressive entre deux espèces de *Sarotherodon* (Pisces, Cichlidae) dans un lac de Madagascar. Bulletin du Museum National d′Histoire Naturelle 4: 689–703.

[51] Weyl OLF (2008) Rapid invasion of a subtropical lake fishery in central Mozambique by Nile tilapia, *Oreochromis niloticus* (Pisces: Cichlidae). Aquatic Conservation-Marine and Freshwater Ecosystems 18: 839–851.

[52] Crispo E, Chapman LJ (2010) Temporal variation in population genetic structure of a riverine African cichlid fish. Journal of Heredity 101: 97–106. 19734258 10.1093/jhered/esp078

[53] Zengeya TA, Robertson MP, Booth AJ, Chimimba CT (2012) A qualitative ecological risk assessment of the invasive Nile tilapia, *Oreochromis niloticus* in a sub-tropical African river system (Limpopo River, South Africa). Aquatic Conservation: Marine and Freshwater Ecosystems 23: 51–64.

[54] Zengeya T, Booth A, Bastos A, Chimimba C (2011) Trophic interrelationships between the exotic Nile tilapia, *Oreochromis niloticus* and indigenous tilapiine cichlids in a subtropical African river system (Limpopo River, South Africa). Environmental Biology of Fishes 92: 479–489.

[55] Hohenlohe PA, Bassham S, Etter PD, Stiffler N, Johnson EA, et al (2010) Population Genomics of Parallel Adaptation in Threespine Stickleback using Sequenced RAD Tags. PLoS Genet 6: e1000862. 20195501 10.1371/journal.pgen.1000862 PMC2829049

[56] Agnèse JF, Adepo-Gourene B, Nyingi D (2008) Functional microsatellite and possible selective sweep in natural populations of the black-chinned tilapia *Sarotherodon melanotheron* (Teleostei, Cichlidae). Marine Genomics 1: 103–107. 21798160 10.1016/j.margen.2008.10.004

[57] Baird NA, Etter PD, Atwood TS, Currey MC, Shiver AL, et al (2008) Rapid SNP Discovery and Genetic Mapping Using Sequenced RAD Markers. PLoS ONE 3: e3376. 18852878 10.1371/journal.pone.0003376 PMC2557064

[58] Miller MR, Dunham JP, Amores A, Cresko WA, Johnson EA (2007) Rapid and cost-effective polymorphism identification and genotyping using restriction site associated DNA (RAD) markers. Genome Research 17: 240–248. 17189378 10.1101/gr.5681207 PMC1781356

[59] McKinna EM, Nandlal S, Mather PB, Hurwood DA (2010) An investigation of the possible causes for the loss of productivity in genetically improved farmed tilapia strain in Fiji: inbreeding versus wild stock introgression. Aquaculture Research 41: e730–e742.

[60] Pullin R (1988) Tilapia Genetic Resources for Aquaculture; Management ICfLAR, editor. Manila, Philippines.

[61] Agustin LQ, Mather PB, Wilson JC (1997) Levels and patterns of genetic diversity in *Oreochromis mossambicus*: Wild African *vs.* introduced feral populations in the Australasian/Pacific region. In: Fitzsimmons K, editor. Tilapia Aquaculture: Northeast Regional Agricultural Engineering Service,Ithaca, NY. pp. 75–86.

[62] Bezault E, Balaresque P, Toguyeni A, Fermon Y, Araki H, et al (2011) Spatial and temporal variation in population genetic structure of wild Nile tilapia (*Oreochromis niloticus*) across Africa. BMC Genetics 12: 102. 22151746 10.1186/1471-2156-12-102 PMC3260159

[63] Hulata G, Karplus I, Harpaz S (1995) Evaluation of some red tilapia strains for aquaculture: growth and colour segregation in hybrid progeny. Aquaculture Research 26: 765–771.

[64] Watanabe WO, Fitzsimmons K, Yi Y (2006) Farming tilapia in saline water. In: Lim C, Webster, C.D., editor. Tilapia: Biology, Culture and Nutrition:The Haworth Press, Inc.

[65] Simler KR, Nhate V (2005) Poverty, inequality, and geographic targeting: Evidence from Small-Area Estimates in Mozambique. In: International Food Policy Research Institute, editor.FCND discussion papers.

[66] Bezault E, Rognon X, Gharbi K, Baroiller J-F, Chevassus B (2012) Microsatellites Cross-Species Amplification across Some African Cichlids. International Journal of Evolutionary Biology 2012: 7. 10.1155/2012/870935 PMC3373121 22701809

[67] Nagl S, Tichy H, Mayer WE, Samonte IE, McAndrew BJ, et al (2001) Classification and phylogenetic relationships of African tilapiine fishes inferred from mitochondrial DNA sequences. Molecular Phylogenetics and Evolution 20: 361–374. 11527464 10.1006/mpev.2001.0979

[68] Klett V, Meyer A (2002) What, if anything, is a Tilapia? - Mitochondrial ND2 phylogeny of tilapiines and the evolution of parental care systems in the African cichlid fishes. Molecular Biology and Evolution 19: 865–883. 12032243 10.1093/oxfordjournals.molbev.a004144

[69] Vos P, Hogers R, Bleeker M, Reijans M, Vandelee T, et al (1995) AFLP - a new techique for DNA-fingerprinting. Nucleic Acids Research 23: 4407–4414. 7501463 10.1093/nar/23.21.4407 PMC307397

[70] Herder F, Pfaender J, Schliewen UK (2008) Adaptive sympatric speciation of polychromatic "roundfin" sailfin silverside fish in Lake Matano (Sulawesi). Evolution 62: 2178–2195. 18616575 10.1111/j.1558-5646.2008.00447.x

[71] Bonin A, Bellemain E, Eidesen PB, Pompanon F, Brochmann C, et al (2004) How to track and assess genotyping errors in population genetics studies. Molecular Ecology 13: 3261–3273. 15487987 10.1111/j.1365-294X.2004.02346.x

[72] Pompanon F, Bonin A, Bellemain E, Taberlet P (2005) Genotyping errors: Causes, consequences and solutions. Nature Reviews Genetics 6: 847–859. 10.1038/nrg1707 16304600

[73] Guillot G, Carpentier-Skandalis A (2011) On the informativeness of dominant and co-dominant genetic markers for Bayesian supervised clustering. The Open Statistics and Probability Journal 3: 7–12.

[74] Thompson JD, Higgins DG, Gibson TJ (1994) CLUSTAL W: improving the sensitivity of progressive multiple sequence alignment through sequence weighting, position-specific gap penalties and weight matrix choice. Nucleic Acids Research 22: 4673–4680. 7984417 10.1093/nar/22.22.4673 PMC308517

[75] Hall TA (1999) BioEdit: a user-friendly biological sequence alignment editor and analysis program for Windows 95/98/NT. Nucleic Acids Symposium Series 41: 95–98.

[76] Templeton AR, Crandall KA, Sing CF (1992) A cladistic analysis of phenotypic association with haplotypes inferred from restriction endonuclease mapping and DNA sequence data. III. Cladogram estimation. Genetics 132: 619–635. 1385266 10.1093/genetics/132.2.619 PMC1205162

[77] Paradis E (2010) pegas: an R package for population genetics with an integrated-modular approach. Bioinformatics 26: 419–420. 20080509 10.1093/bioinformatics/btp696

[78] R Development Core Team (2008) R: A language and environment for statistical computing. R Foundation for Statistical Computing, Vienna, Austria ISBN 3-900051-07-0. Available: http://www.R-project.org.Accessed July 2012.

[79] Nei M (1987) Molecular evolutionary genetics. New York: Columbia University Press.

[80] Excoffier L, Smouse PE, Quattro JMA (1992) Analysis of molecular variance inferred from metric distances among DNA haplotypes: application to human mitochondrial DNA restriction data. Genetics 131: 479–491. 1644282 10.1093/genetics/131.2.479 PMC1205020

[81] Vekemans X, Beauwens T, Lemaire M, Roldan-Ruiz I (2002) Data from amplified fragment length polymorphism (AFLP) markers show indication of size homoplasy and of a relationship between degree of homoplasy and fragment size. Molecular Ecology 11: 139–151. 11903911 10.1046/j.0962-1083.2001.01415.x

[82] Zhivotovsky LA (1999) Estimating population structure in diploids with multilocus dominant DNA markers. Molecular Ecology 8: 907–913. 10434412 10.1046/j.1365-294x.1999.00620.x

[83] Pritchard JK, Stephens M, Donnelly P (2000) Inference of population structure using multilocus genotype data. Genetics 155: 945–959. 10835412 10.1093/genetics/155.2.945 PMC1461096

[84] Falush D, Stephens M, Pritchard JK (2007) Inference of population structure using multilocus genotype data: dominant markers and null alleles. Molecular Ecology Notes 7: 574–578. 18784791 10.1111/j.1471-8286.2007.01758.x PMC1974779

[85] Hubisz MJ, Falush D, Stephens M, Pritchard JK (2009) Inferring weak population structure with the assistance of sample group information. Molecular Ecology Resources 9: 1322–1332. 21564903 10.1111/j.1755-0998.2009.02591.x PMC3518025

[86] Evanno G, Regnaut S, Goudet J (2005) Detecting the number of clusters of individuals using the software STRUCTURE: a simulation study. Molecular Ecology 14: 2611–2620. 15969739 10.1111/j.1365-294X.2005.02553.x

[87] Anderson EC (2008) Bayesian inference of species hybrids using multilocus dominant genetic markers. Philosophical Transactions of the Royal Society B-Biological Sciences 363: 2841–2850. 10.1098/rstb.2008.0043 PMC2606736 18508754

[88] Anderson EC, Thompson EA (2002) A model-based method for identifying species hybrids using multilocus genetic data. Genetics 160: 1217–1229. 11901135 10.1093/genetics/160.3.1217 PMC1462008

[89] Kumar S, Skjaeveland A, Orr R, Enger P, Ruden T, et al (2009) AIR: A batch-oriented web program package for construction of supermatrices ready for phylogenomic analyses. BMC Bioinformatics 10: 357. 19863793 10.1186/1471-2105-10-357 PMC2777179

