# Correction: Successive Invasion-Mediated Interspecific Hybridizations and Population Structure in the Endangered Cichlid *Oreochromis mossambicus*

**DOI:** 10.1371/annotation/6a9a9d59-8c24-4c3b-b125-2efba2db4d45

**Published:** 2013-10-25

**Authors:** Cyril Firmat, Paul Alibert, Michèle Losseau, Jean-François Baroiller, Ulrich K. Schliewen

The current Figure S1 is a duplicate of Figure 1. Please see the correct FigureS1 here: 
