# Supplementary material for: Successive Invasion-Mediated Interspecific Hybridizations and Population Structure in the Endangered Cichlid Oreochromis mossambicus
Source: PLoS One. 2013 May 9;8(5):e63880. doi: 10.1371/journal.pone.0063880 (PMC3650077; doi:10.1371/journal.pone.0063880)

**Figure S2. STRUCTURE analysis of the AFLP dataset comprising only individuals with no detected *O. niloticus* component.**

**Figure S2-A.** Averaged log probability of the data Ln P($X|K$) (upper panel) and the value of the $\Delta K$ criteria (lower panel) computed according to Evanno *et al.* (2005) for each number of cluster $K$.

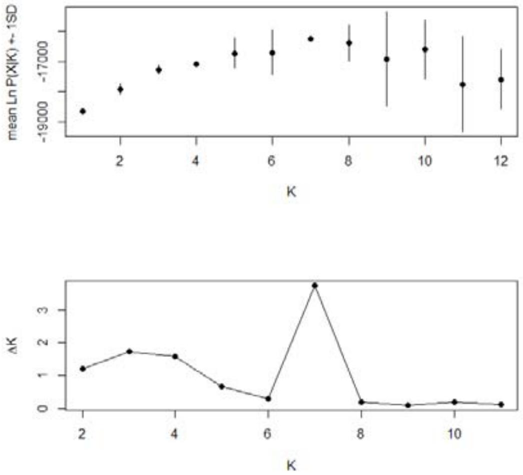


**Figure S2-B.** STRUCTURE barplots for $K$ = 2 to 7 showing assignment values ($Q$) of individuals with no *O. niloticus* component.

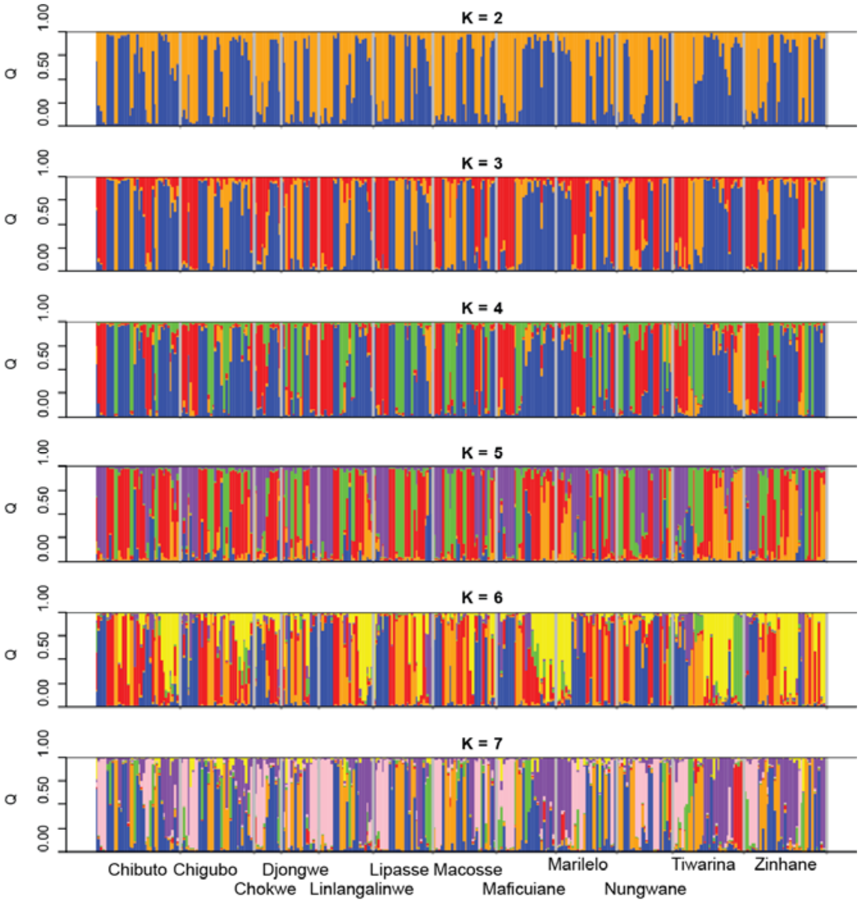

Supplement: Figure S2 — STRUCTURE analysis of the AFLP dataset comprising only individuals with no detected O. niloticus component. A. Averaged log probability of the data Ln P(X|K) (upper panel) and the value of the ΔK criteria (lower panel) computed according to Evanno et al. (2005) for each number of cluster K. B. STRUCTURE barplots for K = 2 to 7 showing assignment values (Q) of individuals with no O. niloticus component. (PDF) [file pone.0063880.s002.pdf]
