# Supplementary material for: Successive Invasion-Mediated Interspecific Hybridizations and Population Structure in the Endangered Cichlid Oreochromis mossambicus
Source: PLoS One. 2013 May 9;8(5):e63880. doi: 10.1371/journal.pone.0063880 (PMC3650077; doi:10.1371/journal.pone.0063880)

Figure S3. **STRUCTURE analysis of the AFLP dataset only comprising *O. mossambicus* individuals from the eight localities preserved from genetic introgression.**

Figure S3-A. Averaged log probability of the data Ln *P*(*X*|*K*) (upper panels) and the value of the *ΔK* criteria (lower panels) computed according to Evanno et al. (2005) for each number of cluster *K*, with (left) and without (right) the LOCPRIOR option.

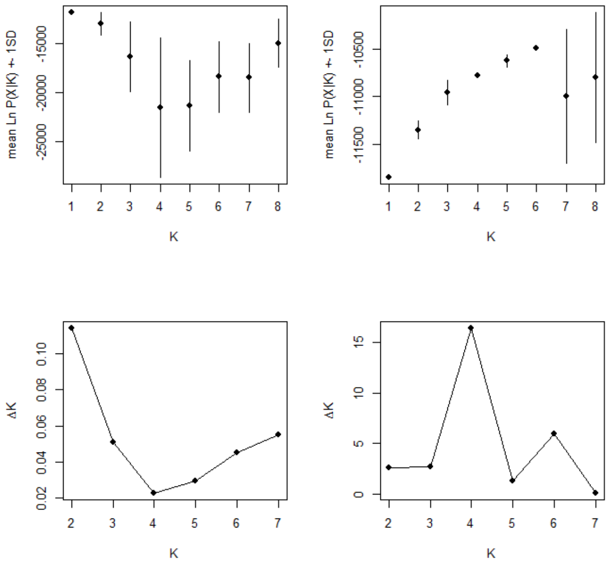

Figure S3-B. STRUCTURE barplots for *K* = 2 to 6 showing the assignment values (*Q*) of *O. mossambicus* individuals from the eight localities preserved from genetic introgressions, with (left) and without (right) the LOCPRIOR option.

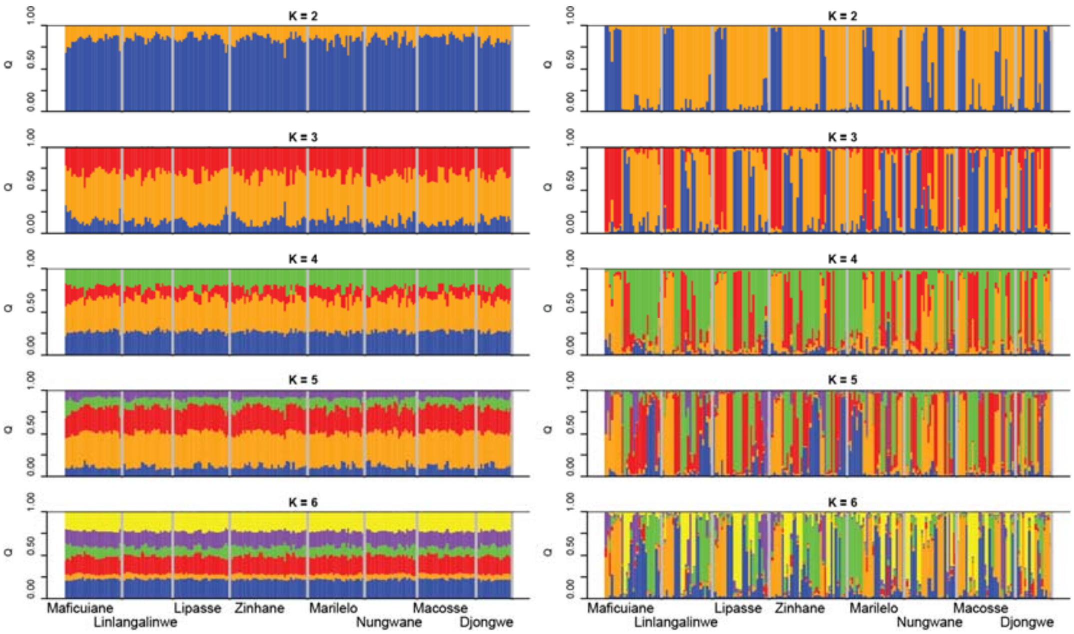

Supplement: Figure S3 — STRUCTURE analysis of the AFLP dataset only comprising O. mossambicus individuals from the eight localities preserved from genetic introgression. A. Averaged log probability of the data Ln P(X|K) (upper panels) and the value of the ΔK criteria (lower panels) computed according to Evanno et al. (2005) for each number of cluster K, with (left) and without (right) the LOCPRIOR option. B. STRUCTURE barplots for K = 2 to 6 showing the assignment values (Q) of O. mossambicus individuals from the eight localities preserved from genetic introgressions, with (left) and without (right) the LOCPRIOR option. (PDF) [file pone.0063880.s003.pdf]
