# Supplementary material for: Successive Invasion-Mediated Interspecific Hybridizations and Population Structure in the Endangered Cichlid Oreochromis mossambicus
Source: PLoS One. 2013 May 9;8(5):e63880. doi: 10.1371/journal.pone.0063880 (PMC3650077; doi:10.1371/journal.pone.0063880)

Figure S4. Haplotype genealogy of the genus *Oreochromis* based on a 385 bp fragment of the mitochondrial control region. The size of the circles representing each haplotype is proportional to log($N_{individuals}$).

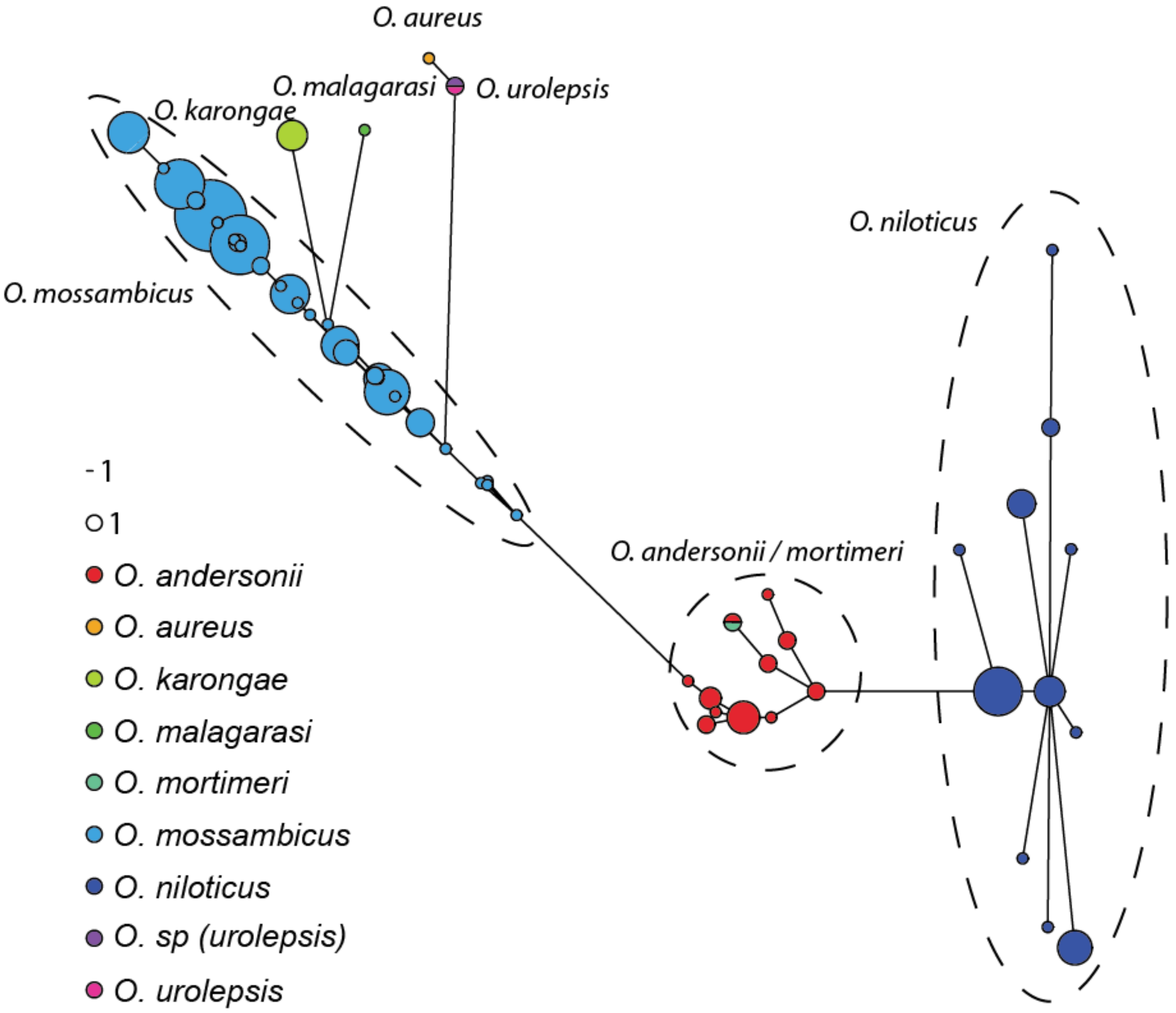

Supplement: Figure S4 — Haplotype genealogy of the genus Oreochromis based on a 385 bp fragment of the mitochondrial control region. The size of the circles representing each haplotype is proportional to log(N individuals). (PDF) [file pone.0063880.s004.pdf]
