# Supplementary material for: Successive Invasion-Mediated Interspecific Hybridizations and Population Structure in the Endangered Cichlid Oreochromis mossambicus
Source: PLoS One. 2013 May 9;8(5):e63880. doi: 10.1371/journal.pone.0063880 (PMC3650077; doi:10.1371/journal.pone.0063880)
Supplement: Table S2 — Number of AFLP loci per primer combination. (PDF) [file pone.0063880.s006.pdf]

Table S2. Number of AFLP loci per primer combination.

EcoRI-ACA/MseI-CAA	EcoRI-ACA/MseI-CTT	EcoRI-ACT/MseI-CTC	EcoRI-ACC/MseI-CTG	EcoRI-ACT/MseI-CTG	EcoRI-ACT/MseI-CAC
89	83	56	62	67	66
