# Supplementary material for: Successive Invasion-Mediated Interspecific Hybridizations and Population Structure in the Endangered Cichlid Oreochromis mossambicus
Source: PLoS One. 2013 May 9;8(5):e63880. doi: 10.1371/journal.pone.0063880 (PMC3650077; doi:10.1371/journal.pone.0063880)
Supplement: Table S3 — Control region sequences from GenBank used in this study. Sequences marked with * were included in the haplotypes networks of the figures 3 and 4. (PDF) [file pone.0063880.s007.pdf]

Table S3. Control region sequences from GenBank used in this study.

Sequences marked with * were included in the haplotypes networks of the figures 3 and 4.

GenBank accession Number	Species
AY833435	*O. urolepsis*
AY833436	*O. mossambicus*
AY833437	*O. mossambicus*
AY833438	*O. mossambicus*
AY833439	*O. mossambicus*
AY833440	*O. mossambicus*
AY833441	*O. mossambicus*
AY833442	*O. mossambicus*
AY833443	*O. mossambicus*
AY833444	*O. mossambicus*
AY833445	*O. mossambicus*
AY833446	*O. mossambicus*
AY833447	*O. mossambicus*
AF328843	*O. mossambicus*
AY833448	*O. mossambicus*
AY833449	*O. mossambicus*
AY833450	*O. mossambicus*
AY833451	*O. mossambicus*
AY833452	*O. mossambicus*
AY833453	*O. mossambicus*
AY833454	*O. mossambicus*
AY833455	*O. mossambicus*
AY833456	*O. mossambicus*
AY833457	*O. mossambicus*
AY833458	*O. mossambicus*
AY833459	*O. mossambicus*
AY833460	*O. karongae*
AY833461	*O. karongae*
AY833462	*O. karongae*
AY833463	*O. karongae*
AY833464	*O. karongae*
AY833465	*O. karongae*
AY833466	*O. niloticus*
AY833467*	*O. niloticus*
AY833468*	*O. niloticus*
AY833469*	*O. niloticus*
AY833470	*O. niloticus*
AY833471	*O. niloticus*
AY833472	*O. niloticus*
AY833473	*O. niloticus*

AF328849	*O. niloticus*
AY833474*	*O. niloticus*
AY833475*	*O. niloticus*
AY833476*	*O. niloticus*
AY833477*	*O. niloticus*
AY833478*	*O. niloticus*
AY833479*	*O. niloticus*
AY833480*	*O. niloticus*
AY833481*	*O. niloticus*
AY833482	*O. niloticus*
AY833483	*O. niloticus*
AY833484	*O. niloticus*
AY833485	*O. niloticus*
AY833486	*O. niloticus*
AY833487	*O. niloticus*
AY833488	*O. niloticus*
AY833489	*O. niloticus*
AY833490	*O. niloticus*
AY833491	*O. niloticus*
AF296488	*O. andersonii*
AY833492*	*O. andersonii*
AY833493*	*O. andersonii*
AY833494*	*O. andersonii*
AY833495*	*O. andersonii*
AY833496*	*O. andersonii*
AY833497*	*O. andersonii*
AY833498*	*O. andersonii*
AY833499*	*O. andersonii*
AY833500*	*O. andersonii*
AY833501*	*O. andersonii*
AY833502*	*O. andersonii*
AF328845	*O. mortimeri*
AF328851	*O. aureus*
