# Supplementary material for: Successive Invasion-Mediated Interspecific Hybridizations and Population Structure in the Endangered Cichlid Oreochromis mossambicus
Source: PLoS One. 2013 May 9;8(5):e63880. doi: 10.1371/journal.pone.0063880 (PMC3650077; doi:10.1371/journal.pone.0063880)
Supplement: Table S1 — Abundance and repartition of haplotypes in the Changane-Lower Limpopo System per locality. (PDF) [file pone.0063880.s005.pdf]

Table S1. Abundance and repartition of haplotypes in the Changane-Lower Limpopo System per locality.

Species	Genbank accession #	Localities											
		CHB	CHG	CHK	DJO	LIN	LIP	MAC	MAF	MAR	NUN	TIW	ZIN
O. mossambicus	JQ907486	0	0	0	1	0	0	0	0	0	0	0	0
	JQ907487	0	0	0	6	0	0	0	0	0	0	0	0
	JQ907488	0	0	0	1	0	0	0	0	0	0	0	0
	JQ907489	0	0	0	1	0	0	0	0	0	0	0	0
	JQ907490	0	0	0	1	0	0	0	0	0	0	0	0
	JQ907491	2	1	0	1	0	0	3	0	1	2	0	1
	JQ907492	6	0	0	0	1	1	0	2	3	3	1	0
	JQ907493	5	5	1	0	5	4	5	3	1	3	8	4
	JQ907494	0	2	1	0	0	6	0	0	0	0	0	1
	JQ907495	4	2	1	0	1	2	0	6	2	1	2	2
	JQ907496	0	0	0	0	1	0	0	0	0	0	0	0
	JQ907497	0	0	0	0	1	0	0	0	0	0	0	0
	JQ907498	0	0	0	0	2	0	0	0	0	0	0	0
	JQ907499	0	0	0	0	1	0	0	0	0	0	0	0
	JQ907500	1	2	0	0	1	0	4	1	0	1	0	3
	JQ907501	0	1	0	0	0	0	0	0	0	0	0	0
	JQ907502	0	0	0	0	0	0	0	0	2	2	0	0
	JQ907503	1	0	0	0	0	0	0	0	0	0	0	0
	JQ907504	2	0	0	0	0	0	0	0	0	0	0	0
	JQ907505	1	0	0	0	0	0	0	0	0	0	0	0
	JQ907506	0	0	0	0	0	0	0	0	0	0	0	1
	JQ907507	0	0	0	0	0	0	0	0	0	0	1	0
O. andersonii	JQ907508	0	0	1	0	0	0	0	0	0	0	0	0
	JQ907509	0	1	3	0	0	0	0	0	0	0	0	0
	JQ907510	0	0	2	0	0	0	0	0	0	0	0	0
	JQ907511	0	0	1	0	0	0	0	0	0	0	0	0
	JQ907512	0	0	1	0	0	0	0	0	0	0	0	0
	JQ907513	1	0	0	0	0	0	0	0	0	0	1	0
O. niloticus	JQ907514	4	0	17	0	0	0	0	0	0	0	0	0
