# Supplementary material for: Correction: Successive Invasion-Mediated Interspecific Hybridizations and Population Structure in the Endangered Cichlid Oreochromis mossambicus
Source: PLoS One. 2013 Oct 25;8(10):10.1371/annotation/6a9a9d59-8c24-4c3b-b125-2efba2db4d45. doi: 10.1371/annotation/6a9a9d59-8c24-4c3b-b125-2efba2db4d45 (PMC3808445; doi:10.1371/annotation/6a9a9d59-8c24-4c3b-b125-2efba2db4d45)

Figure S1. STRUCTURE analysis of the full AFLP dataset.

Averaged log probability of the data Ln P(X|K) (upper panel) and the value of the ΔK criteria (lower panel) computed according to Evanno *et al.* (2005) for each number of cluster K. The ΔK plot clearly supports the presence of two clusters in the data.

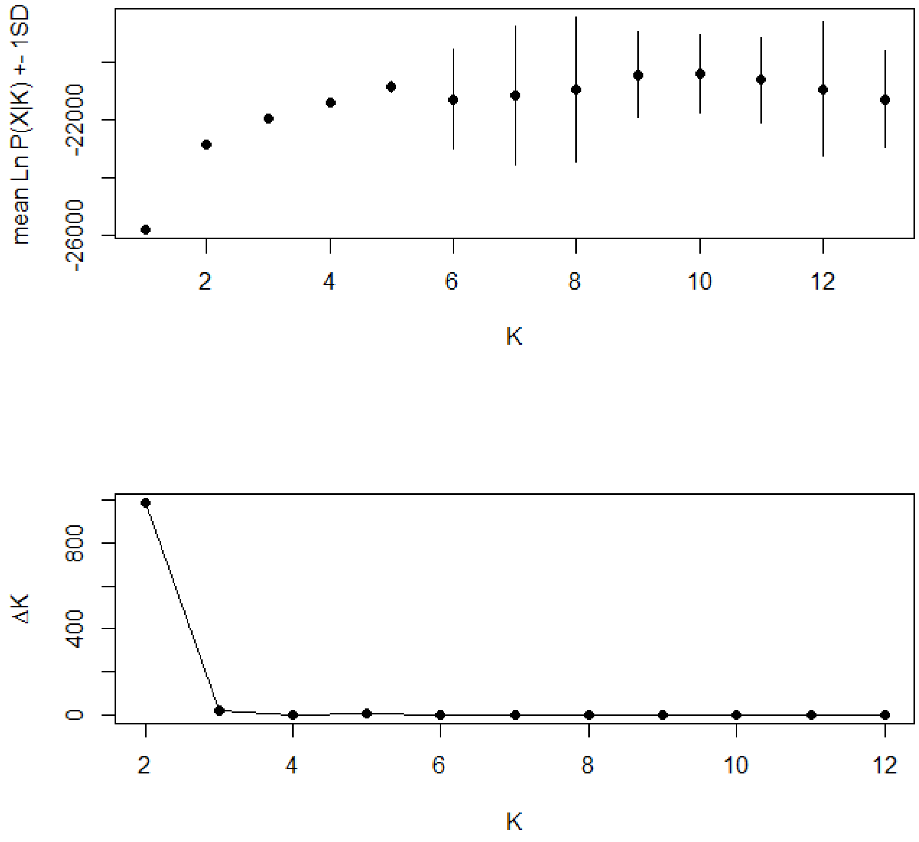

Supplement: Supplementary file 1 [file pone.6a9a9d59-8c24-4c3b-b125-2efba2db4d45.s001.pdf]
